# A Case of Extra-Uterine Pregnancy

**Published:** 1881-05

**Authors:** James P. White


### A CASE OF EXTRA-UTERINE PREGNANCY.—TAPPING OF SAC.—RECOVERY.

BY PROF. JAMES P. WHITE.

REPORTED BY G. H. VAN DEUSEN, M. D., GORHAM, N. Y.

MRS. ——; aged 22; married; one child 2½ years; weakly, delicate organization; consulted me 15th of September, 1880; said she had gone over time; that she menstruated from Aug. 2d to Aug. 8th. I prescribed a preparation of iron, informing her that iron would do her good, and if she were pregnant it would not interfere with it. She said she did not want it interfered with. She was sorry it happened, as she felt she had not strength to take care of children, yet if they came she would do her best to rear them. Oct. 17, I was called to see her for a bilious attack, to which she is subject. She then told me that when she was lying on her right side, and turned over on her left side, a bunch as large as her fist would drop over in the left side, and when she turned on the right side it would fall back; when she stood on her feet a short time she would feel faint; she did not have "morning sickness" during her preceding gestation; made a vaginal examination and found uterus not enlarged and crowded her well over in hollow of sacrum. I failed to find the *lump* she spoke of. There was considerable tenderness in right iliac region. I still advised the iron mixture. December 23d she again called me and desired to know the reason of her regular and scanty menstruation, for the fifth time, lasting only a few hours or a half day and then stopping.

I was so sure she was not pregnant in October that I again made an examination and found uterus just where it was, only lower in vagina; there was no appearance of the fundus above the pubis; the finger in vagina would meet the fingers of the other hand when pressed down above the pubis on the left side; there was a tumor on the right side; I was about to say she was not pregnant, but had an ovarian tumor, when I felt the motions of the child entirely too distinct to be deceived; told her she was pregnant, but child was not in uterus. The "kick" was felt on a line from anterior superior spinous process, of ilium to umbilicus.

Two days later she was taken with labor pains; I was called and found that there was a profuse discharge of mucus, and at each pain the uterus would be forced down farther in the vagina; gave morphine and pains ceased. I corresponded with Prof. J. P. White, in regard to the case.

January 11th, 1881. Prof. White, Dr. Cary and Dr. Granger visited the patient, and severally made a thorough examination, agreeing in the diagnosis of extra uterine pregnancy, for these reasons: The uterine sound only entered two inches farther than in an unimpregnated uterus, and Prof. White satisfied himself beyond a doubt that the uterine cavity was empty; there was a well-developed tumor anterior and above the uterus extending to a level with the umbilicus. The motions of child were felt near the navel, and it seems a physical impossibility for the motions of a fœtus of scarcely five months to be felt so high as that, and the fœtal heart was heard an inch and a half to the right of and below the navel.

The plan of treatment adopted was to tap the sac, draw off the amniotic fluid, thereby arrest the development of fœtus and then wait for future indications for treatment.

A large trochar was introduced into the sac, anteriorly to the uterus and posteriorly to the bladder. There was a discharge of a large quantity of amniotic fluid; patient was then put to bed; there was no shock, though patient suffered from extreme nausea from ether.

January 12th, 8 A. M.; pulse, 104; temperature 101; complaining of severe pain in tumor; gave tr. op. acet., 15 drops every hour. At 12 M. labor pains began; at 4 P. M., pains were intolerable; injected morphine over sac; at 6 P. M., patient got up to use night chair, when she cried out that something was coming from her body, which proved to be a female fœtus of evidently five months' growth; there had been no hemorrhage to call our attention to approaching delivery. At 8 P. M., pulse, 104; temperature 102°.

January 13th, 8 A. M.; pulse, 120; temperature, 103.2°; considerable tenderness over tumor; had slept some under influence chloral hydrat. 8 P. M.; pulse 140; temperature, 104°; very little flowing; no after-pains.

January 14, 8 P. M.; pulse, 120; temperature, 102.8; from this time on she made a satisfactory recovery, and is now enjoying her usual health.

Now, how can we account for this state of things. I omitted to mention in its proper place, that when Prof. White had the sound in uterus and carried the uterus from side to side the tumor moved with it, and suggested that it was possibly a case of interstitial pregnancy. The impregnated ovum, as it passed down the fallopian tube, was arrested just at the opening of the tube in the uterine cavity and there developed, forming its own gestation sac—as it always does, no matter where it is located, whether in the uterus or outside of it—and as it enlarged, it separated the layers of uterine fibres so that the ovum was surrounded by uterine tissue, and as the growth pushed itself upwards and outwards, it formed the lump or bunch of which the patient complained.

Now, when the pains came on and the uterus contracted, the outer layer of fibres were stronger than the inner layer, and these gave way and the fœtus was forced in the uterus cavity and then expelled as any other fœtus would have been. I feel thoroughly satisfied in my own mind that tapping the sac, letting out the amniotic fluid, added greatly to the successful termination of the case, as the outer layers had contracted down around the fœtus, thereby rendering them much thicker and firmer; furthermore, if the case had not been operated on or had been left to take the chances until term, when pains came on the walls would have been just as likely to have ruptured in the abdominal cavity as in the uterine cavity, and then our patient would have died.

